# *HDAC9* Variant Rs2107595 Modifies Susceptibility to Coronary Artery Disease and the Severity of Coronary Atherosclerosis in a Chinese Han Population

**DOI:** 10.1371/journal.pone.0160449

**Published:** 2016-08-05

**Authors:** Xue-bin Wang, Ya-di Han, Shrestha Sabina, Ning-hua Cui, Shuai Zhang, Ze-jin Liu, Cong Li, Fang Zheng

**Affiliations:** 1 Center for Gene Diagnosis, Zhongnan Hospital of Wuhan University, Wuhan, Hubei, China; 2 Department of Clinical Laboratory, Children's Hospital of Zhengzhou, Zhengzhou, Henan, China; 3 Center of Clinical Laboratory, Wuhan Asia Heart Hospital, Wuhan, Hubei, China; 4 Zhongshan School of Medicine, Sun Yat-sen University, Guangzhou, China; Centro Cardiologico Monzino, ITALY

## Abstract

A previous genome-wide association study showed that a single nucleotide polymorphism (SNP) rs2107595 in histone deacetylase 9 (*HDAC9*) gene was associated with large artery stroke (LAS) in Caucasians. Based on the similar atherosclerotic pathogenesis between LAS and coronary artery disease (CAD), we aimed to evaluate the associations of SNP rs2107595 with CAD risk and the severity of coronary atherosclerosis in a Chinese Han population, and explore the potential gene-environment interactions among SNP rs2107595 and conventional CAD risk factors. In a two-stage case-control study with a total of 2317 CAD patients and 2404 controls, the AG + AA genotypes of SNP rs2107595 were significantly associated with increased CAD risk (Adjusted odds ratio (OR) = 1.23, P_adj_ = 0.001) and higher modified Gensini scores (Adjusted OR = 1.38, P_adj_ < 0.001). These associations remained significant in subtype analyses for unstable angina pectoris (UAP), non-ST-segment elevation myocardial infarction (NSTEMI) and ST-segment elevation myocardial infarction (STEMI). Subgroup and multifactor dimensionality reduction analyses (MDR) further found the gene-environment interactions among SNP rs2107595, body mass index, type 2 diabetes and hyperlipidemia in CAD risk and the severity of coronary atherosclerosis. Moreover, patients with CAD had higher levels of *HDAC9* mRNA expression and plasma HDAC9 than controls. Subsequent genotype-phenotype analyses observed the significant correlations of SNP rs2107595 with *HDAC9* mRNA expression and plasma HDAC9 levels in controls and patients with NSTEMI and STEMI. Taken together, our data suggest that SNP rs2107595 may contribute to coronary atherosclerosis and CAD risk through a possible mechanism of regulating *HDAC9* expression and gene-environment interactions.

## Introduction

Coronary artery disease (CAD), the leading cause of death and disability worldwide[1], is mainly caused by multiple interactions between genetic and environmental risk factors of atherosclerosis[2]. Atherosclerosis in the coronary arteries, is a continuous process of deposition of lipoproteins that results in the formation of atherosclerotic plaques, the progression of plaque instability, and eventually myocardial infarction (MI)[3, 4]. Therefore, the genetic and environmental determinants involved in CAD risk may vary across different stages of atherosclerosis, leading to the underlying heterogeneity between stable and unstable CAD[4, 5]. Moreover, this atherosclerotic pathogenesis also applies to the cerebral vessels and contributes to the development of ischemic stroke (IS), especially to the development of large artery stroke (LAS)[6]. Numerous studies have shown that CAD and LAS shared several conventional risk factors and susceptible loci[7, 8], such as smoking, hypertension and single nucleotide polymorphisms (SNPs) on chromosome 9p21, suggesting shared heritability for both diseases[6].

Recently, a genome-wide association study (GWAS) first reported that SNP rs2107595 in 3'-region of histone deacetylase 9 (*HDAC9*) gene was associated with LAS risk[9]. *HDAC9* gene is located on chromosome 7p21.1, and encodes a member of histone decetylases, which is mainly responsible for the deacetylation of histones and subsequent gene transcription[10]. Accumulated evidence highlights the crucial roles of SNP rs2107595 and *HDAC9* gene in the development of atherosclerosis. First, in a population-based cohort study, the minor allele of SNP rs2107595 was correlated with higher carotid intima-media thickness (CIMT)[11], which has been reported as a predictive marker for atherosclerosis[12]. Second, *HDAC9* plays central roles in heart development[13], and is highly expressed in atherosclerotic plaques at human systemic arteries[11]. Finally, *HDAC9* deletion could lead to down-regulation of inflammatory genes[14], up-regulation of lipid-associated genes[14], and dramatic reduction of atherosclerotic lesion size in mice[15]. Taking all these findings together, we hypothesize that SNP rs2107595 may also contribute to the development of coronary atherosclerosis and CAD risk by modifying *HDAC9* expression.

Thus, in this two-stage case-control study, we assessed the associations of SNP rs2107595 with CAD risk and the severity of coronary atherosclerosis, and further performed a genotype-phenotype analysis by detecting *HDAC9* mRNA expression and plasma HDAC9 levels, followed by subgroup and multifactor dimensionality reduction (MDR) analyses to explore the potential gene-environment interactions. This is the first study to assess the associations of SNP rs2107595 with CAD risk and the severity of coronary atherosclerosis in the Chinese population.

## Materials and Methods

### Study population

This two-stage case-control study included a total of 2317 CAD patients and 2404 age- and sex-matched controls. For reducing the impact of population stratification, we only recruited unrelated ethnic Han Chinese in Hubei Province, central China. In the discovery set (Study 1), 1172 patients and 1086 controls were enrolled from Wuhan Asia Heart Hospital between March 2011 and June 2016. The replication set (Study 2) with 1145 cases and 1318 controls was recruited from Zhongnan Hospital of Wuhan University between July 2013 and June 2016. In these two sets, CAD was angiographically defined as stenosis of more than 50% in at least one major coronary artery or their main branches. Then, according to the American College of Cardiology/American Heart Association Task Force on Practice Guidelines[16–18], CAD patients were further categorized based on the following clinical presentation: (1) stable angina pectoris (SAP); (2) unstable angina pectoris (UAP); (3) non-ST-segment elevation MI (NSTEMI); (4) ST-segment elevation MI (STEMI)(S1 Appendix). Finally, for each case, modified Gensini scores[19] were calculated by two independent angiographers to assess the severity of coronary atherosclerosis. The control groups included participants without lumen stenosis validated by coronary angiography and healthy subjects without cardiovascular diseases identified by physical examinations. The following data for each participant were collected: (1) clinical information such as blood pressure, body mass index (BMI), fasting plasma glucose (FPG) and lipid levels; (2) conventional CAD risk factors including smoking status, alcohol drinking status, and histories of type 2 diabetes mellitus (T2DM), hyperlipidemia, and hypertension (S1 Appendix). The following subjects were excluded: (1) individuals with systemic diseases such as cerebrovascular diseases, cancers, autoimmune diseases and renal or hepatic diseases; (2) subjects with cardiac diseases including myocardial bridge, coronary artery spasm and congenital or valvular heart diseases. This study followed the Declaration of Helsinki, and was approved by the Ethics Committees of Wuhan Asia Heart Hospital and Zhongnan Hospital of Wuhan University. Written informed consent was obtained from all participants.

### Criterion of modified Gensini scores

In the modified Gensini scoring system[19], angiographic stenosis of each coronary segment was first scored according to the degree of luminal narrowing: 1 for 0–25% stenosis, 2 for 26–50%, 4 for 51–75%, 8 for 76–90%, 16 for 91–99% and 32 for 100%. Then a multiplier was assigned to each segment depending on the physical importance of the area supplied by that segment: 5 for main left coronary artery (MLCA), 2.5 for proximal left anterior descending coronary artery (LAD) and proximal left circumflex branch, 1.5 for mid-segment of LAD, 0.5 for second diagonal branch and posterolateral branch, and 1 for other branches. Finally, the weighted scores for each segment were added to give modified Gensini scores. As acute coronary occlusion mainly occurs in a previous angiographically non-critical lesion, acute total occlusion was scored as a non-critical lesion (0–5 score) in the modified Gensini scoring system [19].

### SNP rs2107595 genotyping

Genomic DNA of peripheral blood leukocytes was isolated by a phenol/chloroform method. SNP rs2107595 was genotyped by high resolution melting (HRM) analyses using a LightScanner 96 system (Idaho Technology, Salt Lake City, UT, USA)[20, 21], as described in S1 Table. Repeated assays and DNA sequence analysis were used to validate the accuracy of HRM genotyping (S1 Fig).

### Reverse-transcription quantitative PCR analysis of *HDAC9* mRNA

Total RNA was extracted from peripheral blood leukocytes using the Trizol reagent (Invitrogen, Carlsbad, CA, USA). After eliminating DNA contamination using the RNase-Free gDNA eraser, 1μg of total RNA was prepared for reverse transcription using a reverse transcriptase kit (Takara Bio Inc, Kusatsu, Shiga, Japan).Then, to determine *HDAC9* mRNA expression, the cDNA products were used for reverse-transcription quantitative PCR (RT-qPCR) analysis with the SYBR-Green kit on a CFX96 Touch system(Bio-rad, Hercules, CA, USA). RT-qPCR analysis for each sample was performed in triplicate and followed the MIQE guidelines [22]. The relative expression of *HDAC9* was normalized to the expression of reference gene (*GAPDH*) using the 2 ^-△△Cq^ method[23]. Primer sequences and RT-qPCR conditions for *HDAC9* and *GAPDH* were also summarized in S1 Table.

### Assessment of plasma HDAC9 activity

Plasma samples were separated by centrifugation (at 2000 *g* for 10 minutes at 4°C) and stored at -80°C until detection. According to the manufacturer' s instructions, plasma HDAC9 levels were measured by an enzyme-linked immunosorbent assay (ELISA) (HDAC9 ELISA kit, Xinfan Biosystems, Shanghai, China),followed by quantification using a standard cure with the detection limit of 0.1 ng/mL.CV values for intra- and inter-assays were 4.7% and 6.5%, respectively.

### Statistical analyses

For clinical characteristics, the differences in categorical and continuous variables between cases and controls were analyzed by the Pearson ^2^ test and the Student's t-test, respectively. For SNP rs2107595, Hardy-Weinberg equilibrium (HWE) was assessed by the Pearson ^2^ test. Allelic and genotypic association analyses were performed by logistic regression analyses with and without adjustment for age, sex, BMI, smoking status, alcohol drinking status and histories of T2DM, hyperlipidemia and hypertension. The homogeneity between the odds ratios (ORs) of two sets was evaluated by the Breslow-Day test. The association of SNP rs2107595 with modified Gensini scores was analyzed by the Mann-Whitney U test and logistic regression analyses. When samples were stratified, the multiplicative likelihood ratio test was carried out to test the possible gene-environment interactions in CAD risk and the severity of coronary atherosclerosis. The differences in *HDAC9* expression and plasma HDAC9 levels between cases and controls, and the associations of SNP rs2107595 with *HDAC9* expression and plasma HDAC9 levels were assessed by analyses of covariates (ANCOVA) after adjusting for covariates. The correlations of *HDAC9* expression and plasma HDAC9 levels with modified Gensini scores were examined by the Spearman correlation test. All these statistical analyses were performed by SPSS 17.0 (SPSS Inc., Chicago, IL, USA) and P values of less than 0.05 (two-sided) were considered as statistically significant.

In association analyses for SNP rs2107595, we performed the Bonferroni correction and Monte-Carlo permutation tests to control for multiple comparisons. The Monte-Carlo permutation test can formally calculated an emprical P value by repeating permutations 100,000 times to randomly redistribute genotype counts of cases and controls [24]. An emprical P value of less than 0.05 was considered as a stable result for multiple comparisons. A statistical power was calculated by PS 3.0 program (Vanderbilt University, Nashville, TN, USA). To further assess the high-order gene-environment interactions in CAD risk and the severity of coronary atherosclerosis, multifactor dimensionality reduction (MDR) analyses were carried out using MDR 2.0 beta 8.4 program (UPenn, Philadelphia, PA, USA) [25]. In brief, this program first constructed all possible combinations of included variables. Then, by using 100-time cross-validation and 1000-time permutation tests, the best n-factor models for predicting CAD risk and the severity of coronary atherosclerosis were found with the maximal cross-validation consistency (CVC) and the optimal testing accuracy. Finally, for these best n-factor models, the interaction entropy graphs were applied to visually depict the univariate effect of each variable and the pairwise interactions. The univariate and pairwise effects were expressed as the percentage of entropy.

## Results

### Characteristics of study population

As summarized in S2 Table, in two cohorts of our study, there were significant differences in blood pressure, BMI, FPG and lipid levels as well as the frequencies of smoking, alcohol drinking, T2DM, hyperlipidemia, and hypertension between cases and controls. The genotype distributions of SNP rs2107595 were in accordance with HWE in two control groups (in discovery and replication sets, P_HWE_ = 0.734 and 0.904, respectively).

### Allelic and genotypic association analyses between SNP rs2107595 and CAD risk

In the discovery set, allelic association analyses showed that the minor allele A of SNP rs2107595 was significantly associated with increased CAD risk (OR = 1.19, P = 0.008).This significant association was further identified in the replication set with an allelic OR of 1.20 and a P value of 0.002. As the Breslow-Day test confirmed the homogeneity of ORs between the two sets (P = 0.864), a meta-analysis of these two cohorts was carried out. And the results indicated that the minor allele of SNP rs2107595 had a 1.19-fold (P = 6.08 × 10^−5^) increased risk of CAD in the merged set. All these associations remained significant after the permutation test for multiple testing and adjustment for covariates (Table 1). Given a minor allele frequency (MAF) of 0.3055 in controls, a MAF of 0.3442 in cases and the type I error of 0.05, the merged sample size could provide a statistical power of 81.0% to detect the association.

**Table 1:** Allelic and genotypic associations of SNP rs2107595 with CAD risk.

Model	Alleles (G/A)/Genotypes ‡§		Without adjustment			With adjustment †	
	Cases, N	Controls, N	OR (95%CI)	P	P_emp_*	OR (95%CI)	P_adj_
Allelic association analyses ‡							
Discovery set	1548/796	1515/657	1.19 (1.05–1.34)	0.008	0.009	1.18 (1.03–1.35)	0.016
Replication set	1491/799	1824/812	1.20 (1.07–1.36)	0.002	0.002	1.16 (1.02–1.31)	0.020
Merged set	3039/1595	3339/1469	**1.19 (1.09–1.30)**	**6.08 × 10**^**−5**^	4.11 × 10^−5^	**1.16 (1.06–1.28)**	**0.001**
Genotypic association analyses §							
Discovery set							
Additive	495/558/119	526/463/97	1.19 (1.05–1.36)	0.007	0.014	1.19 (1.03–1.36)	0.014
Dominant	495/677	526/560	1.29 (1.09–1.52)	0.003	0.003	1.26 (1.05–1.50)	0.011
Recessive	1053/119	989/97	1.15 (0.87–1.53)	0.324	0.533	1.19 (0.88–1.60)	0.257
Replication set							
Additive	481/529/135	632/560/126	1.21 (1.07–1.36)	0.002	0.009	1.16 (1.02–1.32)	0.020
Dominant	481/664	632/686	1.27 (1.08–1.49)	0.003	0.006	1.21 (1.02–1.43)	0.026
Recessive	1010/135	1192/126	1.26 (0.98–1.64)	0.073	0.169	1.21 (0.92–1.58)	0.169
Merged set							
Additive	976/1087/254	1158/1023/223	**1.20 (1.10–1.31)**	**5.22 × 10**^**−5**^	1.60 × 10^−4^	**1.17 (1.07–1.28)**	**0.001**
Dominant	976/1341	1158/1246	**1.28 (1.14–1.43)**	**3.04 × 10**^**−5**^	3.35 × 10^−5^	**1.23 (1.09–1.39)**	**0.001**
Recessive	2063/254	2181/223	1.20 (0.99–1.46)	0.055	0.093	1.19 (0.98–1.45)	0.088

* Emprical P values were obtained from 100,000-time Monte-Carlo permutation test.

^†^ Adjusted OR (95%CI) and P_adj_ values were obtained from logistic regression analyses after adjusting for age, sex, BMI, smoking status, alcohol drinking status and histories of T2DM, hyperlipidemia and hypertension.

^‡^ In allelic association analyses, the major allele G was considered as the reference.

^§^ In genotypic association analyses, additive model = GG/AG/AA; dominant model = GG (Reference)/AA + AG; recessive model = AG + GG (Reference)/AA.

Bold values indicate statistically significant after the Bonferroni correction (P < 0.05/24 ≈ 0.002)

To further explore the inheritance pattern of SNP rs2107595, genotypic association analyses were performed based on additive, dominant and recessive models (Table 1). In both discovery and replication sets, significant associations were identified between SNP rs2107595 and increased CAD risk under both additive and dominant models (Table 1). When we combined these two cohorts, the dominant model was considered as the most significant model with the largest OR of 1.28 and the smallest P value of 3.04 × 10^−5^. After adjusting for covariates, all these associations remained significant (Table 1). In both allelic and genotypic association analyses, all significant results in the merged set were robust enough to withstand the Bonferroni correction (Table 1).

### Subtype and subgroup analyses for associations between SNP rs2107595 and CAD risk

To further evaluate the effects of SNP rs2107595 on different CAD subtypes and the potential gene-environment interactions, subtype and subgroup analyses were performed under a dominant model (Table 2). In subtype analyses, compared with the GG carriers, subjects with the AG + AA genotypes had a 1.32-fold (P = 7.75 × 10^−4^), a 1.53-fold (P = 1.20 × 10^−5^) and a 1.62-fold (P = 9.61× 10^−5^) increased risk of UAP, NSTEMI and STEMI, respectively,. In subgroup analyses, the association between the AG + AA genotypes and increased CAD risk was consistently significant in almost all subgroups, except for individuals with BMI ≤ 25 and participants without T2DM and hyperlipidemia. Furthermore, we observed the multiplicative interactions of SNP rs2107595 with BMI (P_inter_ = 0.009), T2DM (P_inter_ = 0.004) and hyperlipidemia (P_inter_ = 0.001) status in CAD risk. All the above associations were still significant after adjusting for covariates (Table 2). Based on the Bonferroni correction, we also found significant associations in subtype analyses for UAP, NSTEMI and STEMI as well as in subjects with BMI > 25, T2DM and hyperlipidemia (Table 2).

**Table 2:** Subtype and subgroup analyses for the association between SNP rs2107595 and CAD risk.

Variables	SNP rs2107595 (cases/controls, N)		Without adjustment				With adjustment ‡		
	GG	AG + AA	OR (95%CI)	P	P_emp_*	P_inter_ †	OR (95%CI) ^a^	P_adj_	P_inter_ †
CAD subtypes									
SAP	335/1158	345/1246	0.96 (0.81–1.14)	0.614	0.667	-	0.96 (0.80–1.14)	0.612	-
UAP	320/1158	456/1246	**1.32 (1.12–1.56)**	**7.75 × 10**^**−4**^	6.73× 10^−4^		**1.35 (1.14–1.60)**	**5.70× 10**^**−4**^	
NSTEMI	205/1158	338/1246	**1.53 (1.27–1.86)**	**1.20 × 10**^**−5**^	1.00× 10^−5^		**1.43 (1.17–1.75)**	**4.60× 10**^**−4**^	
STEMI	116/1158	202/1246	**1.62 (1.27–2.06)**	**9.61 × 10**^**−5**^	9.25× 10^−5^		**1.59 (1.23–2.04)**	**3.11× 10**^**−4**^	
Age, years									
≤ 60	493/528	667/550	1.30 (1.10–1.54)	0.002	0.002	0.813	1.27 (1.06–1.53)	0.009	0.712
> 60	483/630	674/696	1.26 (1.08–1.48)	0.004	0.004		1.21 (1.02–1.43)	0.026	
Sex									
Male	535/631	743/692	1.27 (1.09–1.48)	0.003	0.004	0.876	1.20 (1.02–1.41)	0.029	0.691
Female	441/527	598/554	1.29 (1.09–1.53)	0.004	0.003		1.26 (1.05–1.50)	0.014	
BMI, kg/m^2^									
≤ 25	532/702	668/791	1.11 (0.96–1.30)	0.164	0.130	0.009	1.05 (0.90–1.24)	0.530	**0.020**
> 25	444/456	673/455	**1.52 (1.27–1.81)**	**3.53 × 10**^**−6**^	5.00 × 10^−6^		**1.57 (1.27–1.94)**	**2.42 × 10**^**−5**^	
Smoking status									
Yes	315/309	490/360	1.34 (1.09–1.64)	0.006	0.006	0.535	1.26 (1.01–1.57)	0.038	0.871
No	661/849	851/886	1.23 (1.07–1.42)	0.003	0.003		1.21 (1.05–1.40)	0.010	
Drinking status									
Yes	307/281	450/304	1.36 (1.09–1.68)	0.006	0.006	0.498	1.33 (1.05–1.67)	0.016	0.452
No	669/877	891/942	1.24 (1.08–1.42)	0.002	0.005		1.19 (1.04–1.38)	0.015	
T2DM									
Yes	263/289	487/325	**1.65 (1.32–2.05)**	**7.47 × 10**^**−6**^	6.14 × 10^−6^	0.004	**1.61 (1.28–2.03)**	**4.39 × 10**^**−5**^	0.004
No	713/869	854/921	1.13 (0.99–1.30)	0.078	0.099		1.10 (0.95–1.27)	0.193	
Hyperlipidemia									
Yes	238/266	443/283	**1.75 (1.39–2.20)**	**1.84 × 10**^**−6**^	4.23 × 10^−6^	0.001	**1.59 (1.25–2.02)**	**1.73 × 10**^**−4**^	0.013
No	738/892	898/963	1.13 (0.99–1.29)	0.079	0.096		1.13 (0.98–1.30)	0.093	
Hypertension									
Yes	582/435	792/461	1.28 (1.08–1.52)	0.004	0.003	0.996	1.25 (1.05–1.48)	0.035	0.832
No	394/723	549/785	1.28 (1.09–1.51)	0.003	0.003		1.21 (1.02–1.43)	0.030	

Abbreviation: N, number; OR (95CI), odds ratio (95% confidence interval); CAD, coronary artery disease; SAP, stable angina pectoris; UAP, unstable angina pectoris; NSTEMI: non-ST-segment elevation myocardial infarction; STEMI: ST-segment elevation myocardial infarction; BMI, body mass index; T2DM, type 2 diabetes mellitus.

* Emprical P values were obtained from 100,000-time Monte-Carlo permutation test.

^†^ P_inter_ values were obtained from the multiplicative likelihood ratio test to assess the interactions between SNP rs2107595 and selected variables in CAD risk.

^‡^ Adjusted OR (95%CI) and P_adj_ values were obtained from logistic regression analyses after adjusting for age, sex, BMI, smoking status, alcohol drinking status and histories of T2DM, hyperlipidemia and hypertension.

Bold values indicate statistically significant after the Bonferroni correction (P < 0.05/56 ≈8.93× 10^−4^).

### Association of SNP rs2107595 with the severity of coronary atherosclerosis

By using a modified Gensini scoring system, we assessed the severity of coronary atherosclerosis in CAD patients and found that patients with the AG + AA genotypes had higher modified Gensini scores than the GG carriers in discovery (P = 0.028), replication (P = 0.001) and merged sets (P = 6.47 × 10^−5^)(Table 3). When we classified CAD patients into two groups according to the median (30) of modified

**Table 3:** Association of SNP rs2107595 with the severity of coronary atherosclerosis in 2317 CAD patients under a dominant model.

Variables	Modified Gensini scores *					Modified Gensini scores ‡ (≤ 30/> 30, N)		With adjustment ‡		
	N	GG	N	AG + AA	P †	GG	AG + AA	OR (95%CI)	P _adj_	P_inter_ §
Study set										
Discovery	549	29.5 (18.0–63.0)	757	34.0 (20.0–71.0)	0.028	276/219	317/360	1.32 (1.04–1.67)	0.025	
Replication	427	30.0 (18.0–71.0)	584	35.0 (19.6–82.9)	0.001	268/213	299/365	1.45 (1.14–1.85)	0.002	
Merged set	**976**	**29.5 (18.0–66.0)**	**1341**	**35.0 (20.0–75.0)**	**6.47 × 10**^**−5**^	544/432	616/725	**1.38 (1.17–1.64)**	**1.68 × 10**^**−4**^	
CAD subtypes										
SAP	335	20.0 (14.0–29.5)	345	19.5 (14.0–28.8)	0.355	266/58	289/67	0.76 (0.51–1.13)	0.172	
UAP	320	20.0 (17.0–30.0)	456	23.0 (18.0–36.0)	0.016	246/75	304/151	1.49 (1.07–2.08)	0.018	
NSTEMI	205	71.0 (45.0–90.5)	338	76.8 (55.4–97.5)	0.041	18/197	12/316	2.22 (1.01–4.87)	0.047	
STEMI	116	73.8 (53.3–93.5)	202	81.5 (54.0–110.6)	0.023	14/102	11/191	2.51 (1.07–5.89)	0.035	
Age, years										
≤ 60	493	28.5 (18.0–62.5)	667	32.0 (20.0–74.0)	0.005	280/213	318/349	1.38 (1.09–1.76)	0.008	0.806
> 60	483	30.0 (18.0–73.5)	674	38.0 (20.0–78.0)	0.004	264/219	298/376	1.40 (1.10–1.77)	0.007	
Sex										
Male	535	30.0 (18.0–71.0)	743	33.0 (20.0–72.0)	0.018	246/195	273/325	1.36 (1.09–1.71)	0.008	0.810
Female	441	29.0 (18.0–63.5)	598	36.0 (19.9–79.6)	0.001	298/237	343/400	1.41 (1.10–1.81)	0.007	
BMI, kg/m^2^										
≤ 25	532	30.0 (19.5–69.0)	668	31.0 (19.5–70.9)	0.227	293/239	330/338	1.16 (0.91–1.46)	0.229	0.043
> 25	**444**	**28.5 (18.0–63.0)**	**673**	**41.0 (20.0–79.5)**	**8.76 × 10**^**−5**^	251/193	286/387	**1.68 (1.32–2.15)**	**3.26 × 10**^**−5**^	
Smoking status										
Yes	315	29.0 (18.0–59.0)	490	34.3 (20.0–77.0)	0.001	183/132	221/269	1.59 (1.19–2.13)	0.002	0.282
No	661	30.0 (18.0–69.0)	851	35.0 (19.5–74.0)	0.017	361/300	395/456	1.29 (1.05–1.59)	0.015	
Drinking status										
Yes	307	29.0 (18.0–71.0)	450	33.5 (19.5–76.5)	0.001	172/135	209/241	1.40 (1.14–1.71)	0.001	0.919
No	669	30.0 (18.0–63.5)	891	35.0 (20.0–74.0)	0.024	372/297	407/484	1.36 (1.01–1.83)	0.045	
T2DM										
Yes	**263**	**30.0 (18.0–71.0)**	**487**	**48.0 (21.0–88.0)**	**1.15 × 10**^**−4**^	138/125	174/313	**1.89 (1.39–2.58)**	**5.48 × 10**^**−5**^	0.013
No	713	29.0 (18.0–65.0)	854	30.0 (19.5–66.0)	0.122	406/307	442/412	1.21 (0.99–1.48)	0.068	
Hyperlipidemia										
Yes	**238**	**29.0 (18.0–66.0)**	**443**	**46.5 (22.5–87.0)**	**1.01 × 10**^**−7**^	137/101	157/286	**2.31 (1.67–3.21)**	**5.40 × 10**^**−7**^	**1.51× 10**^**−4**^
No	738	30.0 (18.0–66.0)	898	30.0 (19.5–67.6)	0.330	407/331	459/439	1.14 (0.94–1.39)	0.197	
Hypertension										
Yes	582	29.0 (18.0–63.5)	792	33.0 (19.5–75.0)	0.006	324/258	374/418	1.32 (1.06–1.64)	0.013	0.473
No	394	30.0 (18.0–70.6)	549	36.0 (20.0–75.5)	0.003	220/174	242/307	1.49 (1.14–1.94)	0.004	

Abbreviation: N, number; OR (95CI), odds ratio (95% confidence interval); SAP, stable angina pectoris; UAP, unstable angina pectoris; NSTEMI: non-ST-segment elevation myocardial infarction; STEMI: ST-segment elevation myocardial infarction; BMI, body mass index; T2DM, type 2 diabetes mellitus.

* Modified Gensini scores are expressed as median (interquartile range) because of the skewed distributions.

^†^ P values were obtained from the Mann-Whitney U test.

^‡^ CAD patients were classified into two groups based on the median (30) of modified Gensini scores, then logistic regression analyses were used to assess the association between SNP rs2107595 and modified Gensini scores after adjusting for age, sex, BMI, smoking status, alcohol drinking status and histories of T2DM, hyperlipidemia and hypertension.

^§^ P_inter_ values were obtained from the multiplicative likelihood ratio test to assess the multiplicative interactions between SNP rs2107595 and selected variables in the severity of coronary atherosclerosis.

Bold values indicate statistically significant after the Bonferroni correction (P < 0.05/54 ≈9.26× 10^−4^).

Gensini scores, we also observed that the variant genotypes (AG + AA) of SNP rs2107595 were associated with a 1.38-fold (P_adj_ = 1.68 × 10^−4^) increased risk of higher modified Gensini scores (> 30) in the merged set after adjusting for covariates. These associations remained significant in almost all subtypes and subgroups, except for SAP subtypes as well as subgroups of BMI ≤ 25, non-T2DM and non-hyperlipidemia (Table 3). Moreover, the AG + AA genotypes multiplicatively interacted with higher BMI (> 25)(P_inter_ = 0.043), and histories of T2DM(P_inter_ = 0.013) and hyperlipidemia(P_inter_ = 1.51× 10^−4^) to increase the severity of coronary atherosclerosis. After the Bonferroni correction, the associations in the merged set as well as in patients with BMI > 25, T2DM and hyperlipidemia remained significant (Table 3).

### MDR analyses for assessing the high-order gene-environment interactions in CAD risk and the severity of coronary atherosclerosis

**Fig 1:**
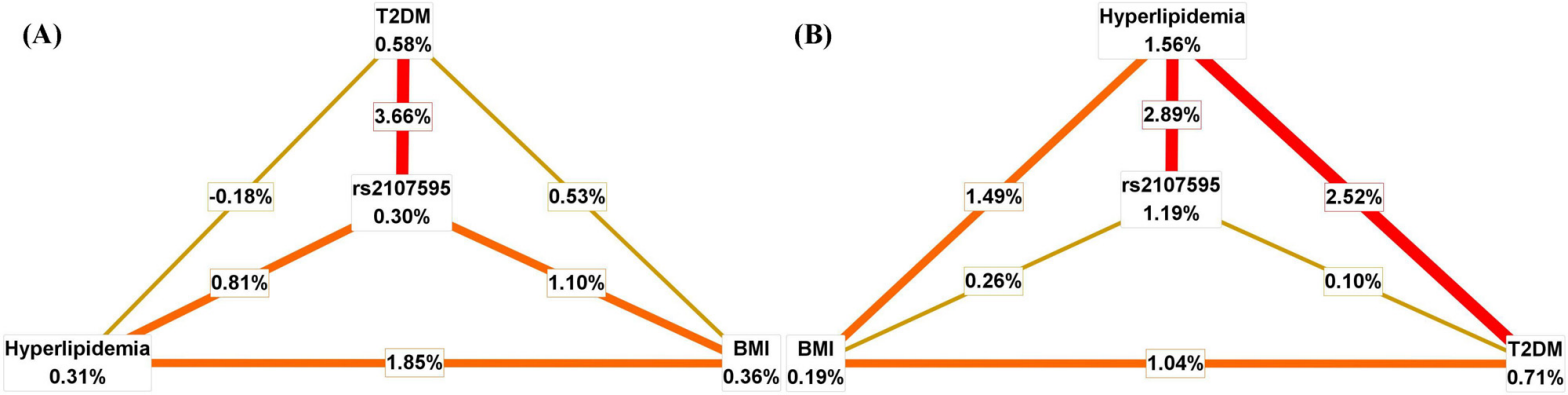
Interaction entropy graphs for the gene-environment interactions between SNP rs2107595, BMI status, and histories of T2DM and hyperlipidemia in CAD risk and the severity of coronary atherosclerosis. (A)Interaction entropy graphs for the gene-environment interactions between SNP rs2107595, BMI status, and histories of T2DM and hyperlipidemia in CAD risk; (B)Interaction entropy graphs for the gene-environment interactions between SNP rs2107595, BMI status, and histories of T2DM and hyperlipidemia in the severity of coronary atherosclerosis. In these graphs, the univariate effect of each variable and the pairwise interactions are expressed as the percentage of entropy. The univariate effect of each variable is presented in a box with the percentage of entropy. The pairwise interactions are shown as connected lines accompanied by the percentage of entropy. Different colors on lines indicate the strength of the interactions, *i*.*e*. red, orange and yellow lines mean strong, moderate and weak interactions, respectively.

Based on the results of multiplicative interaction analyses, data on SNP rs2107595, BMI, T2DM and hyperlipidemia status were included in the MDR analyses to further explore the high-order gene-environment interactions. As presented in Table 4, the four-factor model including all variables was selected as the best predictor for both CAD risk and higher modified Gensini scores, because it had the optimal testing accuracy and the maximal CVC values. For this four-factor model, when evaluating the univariate effects of different variables, history of T2DM (0.58%)exerted the greatest independent effect on CAD risk (Fig 1A), while history of Hyperlipidemia (1.56%) was the strongest risk factor for higher modified Gensini scores (Fig 1B). When assessing the strength of pairwise effects, the strongest pairwise interactions were found between SNP rs2107595 and T2DM (3.66%) in MDR analyses for predicting CAD risk (Fig 1A), and between SNP rs2107595 and hyperlipidemia (2.89%) in MDR analyses for predicting the severity of coronary atherosclerosis (Fig 1B).

**Table 4:** The gene-environment interactions between SNP rs2107595 and conventional CAD risk factors in CAD risk and the severity of coronary atherosclerosis by MDR analyses.

No. of risk factors	Best interaction models	CVC *	Testing accuracy (%) †	P for permutation test ‡
Gene-environment interactions in CAD risk, 2317 CAD patients/2404 controls				
1	History of T2DM	94/100	0.5440	0.0013
2	History of T2DM, SNP rs2107595 (GG/AG + AA)	100/100	0.6211	< 0.0001
3	History of T2DM, SNP rs2107595 (GG/AG + AA), BMI status	100/100	0.6547	< 0.0001
**4**	**History of T2DM, SNP rs2107595 (GG/AG + AA), BMI status, history of hyperlipidemia**	**100/100**	**0.6869**	**< 0.0001**
Gene-environment interactions in the severity of coronary atherosclerosis, 1160/1157 CAD patients with lower (≤ 30)/higher (> 30) modified Gensini scores				
1	History of hyperlipidemia	96/100	0.5691	0.0001
2	History of hyperlipidemia, SNP rs2107595 (GG/AG + AA)	100/100	0.6219	< 0.0001
3	History of hyperlipidemia, SNP rs2107595 (GG/AG + AA), BMI status	100/100	0.6300	< 0.0001
**4**	**History of hyperlipidemia, SNP rs2107595 (GG/AG + AA), BMI status, history of T2DM**	**100/100**	**0.6698**	**< 0.0001**

Abbreviation: T2DM, type 2 diabetes mellitus; BMI, body mass index; CVC, cross-validation consistency.

* CVC means the number of times that a given combination of factors is identified in each testing set (a total of 100 times).

^†^ Testing accuracy (%) is the percentage of participants for whom a correct prediction is made.

^‡^ The permutation test was carried out to repeat the MDR analyses 1000 times and calculate the CVC and testing accuracy of each *n*-factor model.

Bold values indicate the models that have the maximal CVC and the optimal testing accuracy.

### The differences in *HDAC9* mRNA expression and plasma HDAC9 levels between CAD patients and controls

We measured *HDAC9* mRNA expression and plasma HDAC9 levels in 250 representative subjects (125 cases vs 125 controls, S3 Table) randomly selected from the replication set. In ANCOVA models adjusted for covariates, *HDAC9* mRNA expression (1.20 ± 0.41 vs 1.06± 0.39, P = 0.031) and plasma HDAC9 levels (54.2± 9.9 vs 50.6± 8.7, P = 0.015) in CAD patients were significantly higher than those in controls. In subtype analyses, patients with NSTEMI and STEMI had higher levels of *HDAC9* mRNA expression and plasma HDAC9 than controls (Table 5). Moreover, we also observed a positive correlation between plasma HDAC9 levels and modified Gensini scores (r = 0.184, P = 0.040).

**Table 5:** Associations of SNP rs2107595 with *HDAC9* mRNA expression and plasma HDAC9 levels.

	Control		CAD		SAP		UAP		NSTEMI		STEMI	
	N	Mean ± SD	N	Mean ± SD	N	Mean ± SD	N	Mean ± SD	N	Mean ± SD	N	Mean ± SD
*HDAC9* mRNA expression												
Total	125	1.06 ± 0.39	125	1.20± 0.41*	45	1.15 ± 0.38	34	1.15 ± 0.42	25	1.26 ± 0.44*	21	1.34 ± 0.39*
SNP rs2107595												
GG	65	0.97 ± 0.36	50	1.09 ± 0.40	17	1.16 ± 0.38	17	1.15 ± 0.45	10	0.95 ± 0.42	6	0.98 ± 0.29
AG	46	1.13 ± 0.40†	54	1.25 ± 0.36†	22	1.16 ± 0.40	11	1.16 ± 0.35	9	1.41 ± 0.34†	12	1.39 ± 0.25†
AA	14	1.23 ± 0.42†	21	1.34 ± 0.47†	6	1.08 ± 0.35	6	1.13 ± 0.50	6	1.55 ± 0.31†	3	1.85 ± 0.40†
AG + AA	60	1.16 ± 0.40†	75	1.28 ± 0.39†	28	1.14 ± 0.39	17	1.15 ± 0.39	15	1.47 ± 0.32†	15	1.48 ± 0.33†
Plasma HDAC9 levels (ng/mL)												
Total	125	50.6 ± 8.7	125	54.2± 9.9*	45	52.3 ± 10.3	34	53.1 ± 10.4	25	56.6 ± 8.3*	21	57.0 ± 9.1*
SNP rs2107595												
GG	65	48.6 ± 8.2	50	52.3 ± 9.5	17	53.6 ± 8.7	17	52.9 ± 12.0	10	50.7 ± 6.0	6	49.7 ± 9.3
AG	46	51.6 ± 8.8	54	55.4 ± 10.2	22	51.9 ± 11.1	11	54.3 ± 9.8	9	59.8 ± 9.2†	12	59.6 ± 7.8†
AA	14	57.2 ± 7.5†	21	55.3 ± 9.8	6	50.3 ± 13.1	6	51.2 ± 7.8	6	61.6 ± 3.5†	3	60.9 ± 7.1†
AG + AA	60	52.9 ± 8.8†	75	55.4 ± 10.0	28	51.6 ± 11.3	17	53.2 ± 9.0	15	60.5 ± 7.3†	15	59.9 ± 7.5†

Abbreviation: N, number; SD, standard deviation; CAD, coronary artery disease; SAP, stable angina pectoris; UAP, unstable angina pectoris; NSTEMI: non-ST-segment elevation myocardial infarction; STEMI: ST-segment elevation myocardial infarction.

* P < 0.05, in the comparisons between CAD patients and controls.

^†^ P < 0.05, in the comparisons between different genotypes of SNP rs2107595.

### Associations of SNP rs2107595 with *HDAC9* mRNA expression and plasma HDAC9 levels

**Fig 2:**
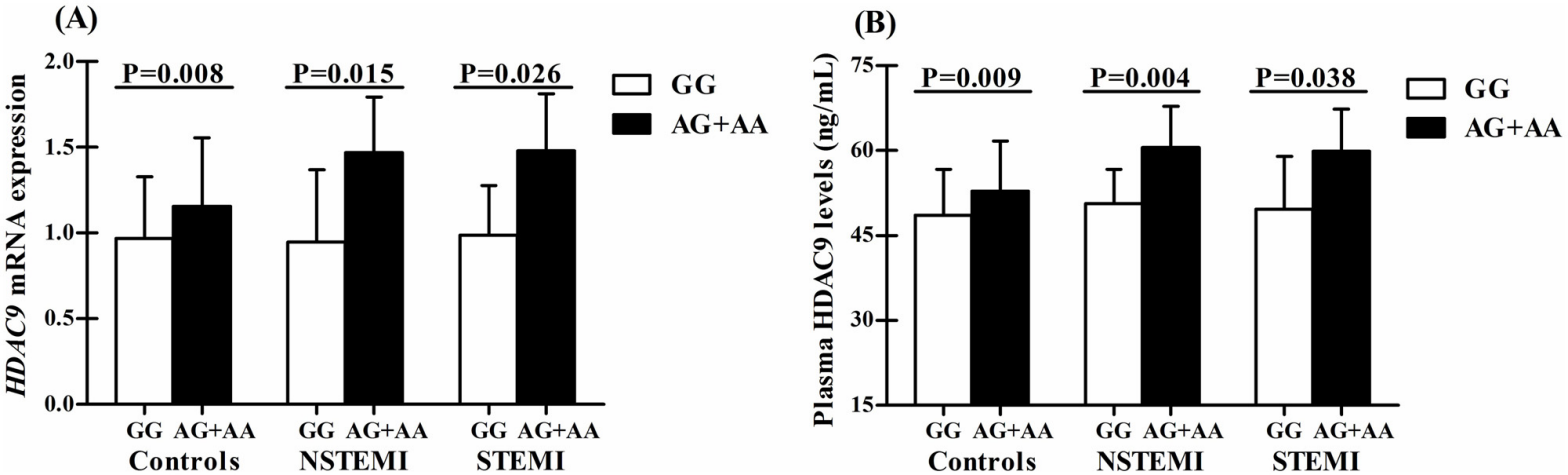
Associations of SNP rs2107595 with *HDAC9* mRNA expression and plasma HDAC9 levels. (A) Associations of SNP rs2107595 with *HDAC9* mRNA expression in controls and patients with NSTEMI and STEMI; (B) Associations of SNP rs2107595 with plasma HDAC9 levels in controls and patients with NSTEMI and STEMI. ANCOVA models are used to assess statistical significance. Data are expressed as mean ± standard deviation (SD).

Assuming a dominant model, significant correlations were found between the AG + AA genotypes and increased *HDAC9* mRNA expression in both cases (P = 0.011) and controls (P = 0.008), and between the AG + AA genotypes and higher plasma HDAC9 levels only in controls(P = 0.009) (Table 5 and Fig 2). When patients were stratified by CAD subtypes, we further observed the significant associations of the AG + AA genotypes with increased levels of *HDAC9* mRNA expression and plasma HDAC9 in patients with NSTEMI and STEMI (Fig 2).

## Discussion

In 2012, the METASTROKE collaboration reported a significant association between SNP rs2107595 of *HDAC 9* gene and LAS risk in Caucasians[9], suggesting an important role of this locus in atherosclerotic stroke. Subsequent GWAS datasets in Europeans identified that SNPs rs11984041 and rs2023938 in this gene were associated with the risk of LAS[26, 27] and CAD[6, 28], respectively. However, based on the data from a previous report[29] and 1000 Genome Database[30] (http://www.1000genomes.org/), these two SNPs in Caucasians are not polymorphic in the Chinese Han population, suggesting that the genetic architecture in *HDAC9* region may differ across ethnicities. More importantly, no previous studies have assessed the effects of SNP rs2107595 on coronary atherosclerosis and subsequent CAD risk. Therefore, in the current study, by using a two-stage case-control design with a total of 2317 CAD patients and 2404 controls, we genotyped SNP rs2107595 and found that the minor allele A of this locus was significantly associated with increased CAD risk and higher modified Gensini scores in a Chinese Han population. Then, by using subgroup and MDR analyses, the gene-environment interactions among the AG + AA genotypes, higher BMI and histories of T2DM and hyperlipidemia were identified to increase CAD risk and the severity of coronary atherosclerosis. Finally, the genotype-phenotype analyses further suggested that SNP rs2107595 might be functional by regulating *HDAC9* mRNA expression and plasma proteins.

The present study, for the first time, reported the significant associations of SNP rs2107595 with CAD risk and the severity of coronary atherosclerosis. These findings are further supported by the current result that the minor allele A of SNP rs2107595 was correlated with higher *HDAC9* mRNA expression and the previous reports that *HDAC9* expression was involved in the development of atherosclerosis[11, 14]. By predicting the potential functions of SNP rs2107595 in the F-SNP database [31] (http://compbio.cs.queensu.ca/F-SNP/), we found that this variant is located in an evolutionarily conserved region, suggesting the importance of its physical location. Subsequent bioinformatics using the MatInspector [32] (http://www.genomatix.de/index.html) and SiteGA databases [33] (http://wwwmgs.bionet.nsc.ru/cgi-bin/mgs/sitega/index.pl) further showed that the minor allele of this locus disrupted the binding sites of several E2F transcription factors, including repressor E2F-4 and -6, which have been reported to repress gene transcription by forming a heterodimeric complex with RB protein[34]. Although the exact mechanism needs to be further elucidated, the above evidence reinforces the possibility that SNP rs2107595 may regulate *HDAC9* expression and protein levels, then contribute to the development of coronary atherosclerosis and CAD risk.

In a GWAS for Caucasians, SNP rs2107595 was first associated with LAS, which was considered as an atherosclerotic subtype of IS[35]. In the current study, we also found stronger associations of this variant with unstable CAD (UAP, NSTEMI and STEMI) than with SAP. In general, the continuum from SAP to unstable CAD mainly results from the progression of plaque vulnerability caused by the aggravated inflammation in plaques[36]. Notably, this inflammatory process can be further induced by the polarized switching of macrophages from an anti-inflammatory M2 phenotype to a proinflammatory M1 state[37–39]. Recently, Cao et al.[14] have reported that *HDAC9* deficiency could decrease the expression of M1 inflammatory genes and promote M2 polarization. More importantly, in the present study, besides the significant association of SNP rs2107595 with *HDAC9* expression, we also found that *HDAC9* mRNA expression in patients with NSTEMI and STEMI was significantly higher than that of controls. Taking all the evidence together, it is reasonable to hypothesize that SNP rs2107595 and *HDAC9* expression may involve the progression of atherosclerosis, and thus exert stronger effects on unstable CAD than on SAP.

The above evidence also pinpoints the importance of subtype analyses when assessing the associations of susceptible loci with chronic, progressive diseases like CAD and IS. Recently, a case-control study by Su et al.[40] failed to find a significant association of SNP rs2107595 with IS in a southern Chinese population. Besides the relatively small sample size of their study (816 cases and 816 controls, a power of 37% and 60% based on the allelic ORs of previous GWAS (OR = 1.13) and the present study (OR = 1.19), respectively) and the genetic heterogeneity between central (a MAF of 0.306 in our controls) and southern Chinese (a MAF of 0.345 in their controls), another important reason for their non-significant results may be that Su et al.'s study did not define IS subtypes for their cases, and therefore could not detect the association of SNP rs2107595 with specific IS subtypes.

In this study, by using subgroup and MDR analyses, we found the gene-environment interactions among SNP rs2107595, T2DM, hyperlipidemia and BMI in CAD risk and the severity of coronary atherosclerosis. Subsequent interaction entropy graphs further observed the greatest pairwise effect of SNP rs2107595 and T2DM on CAD risk, and the strongest pairwise interaction between SNP rs2107595 and hyperlipidemia in higher modified Gensini scores. For T2DM and SNP rs2107595, recent studies have shown that *HDAC9* ablation mice exhibited improved glucose tolerance and insulin tolerance[41], and that *HDAC9* up-regulation enhanced gluconeogenesis *in vitro*[42]. The important roles of *HDAC9* gene in glucose metabolism, combined with the known impact of dysglycemia on CAD risk [43] as well as the significant correlation of SNP rs2107595 with *HDAC9* expression, support that the interaction between SNP rs2107595 and T2DM may greatly increase CAD risk. In clinical studies, hyperlipidemia has long been associated with the severity of coronary atherosclerosis[44, 45]. For *HDAC9* gene, functional studies in mice also suggested that increased *HDAC9* expression could inhibit cholesterol efflux through reduction of histone acetylation at promoters of cholesterol efflux genes[14]. As the first stage of reverse cholesterol transport[46], cholesterol efflux plays crucial roles in lipid metabolism, and is inversely correlated with dyslipidemia[47] and the severity of coronary atherosclerosis[48]. Therefore, by increasing *HDAC9* expression, the minor allele of SNP rs2107595 may inhibit cholesterol efflux, then interact with hyperlipidemia to aggravate coronary atherosclerosis.

In conclusion, by regulating *HDAC9* expression and interacting with T2DM, hyperlipidemia and BMI status, the minor allele A of SNP rs2107595 increased CAD risk and the severity of coronary atherosclerosis. Functional studies are needed to explain the underlying mechanism.

## Supporting Information

S1 Appendix Supplementary materials and methods (Diagnostic criterion of different CAD subtypes and Definition of clinical characteristics). (DOCX)

S1 Dataset Clinical and genetic data in our population. (XLSX)

S2 Dataset Clinical and genetic data of participants that were randomly selected to measure HDAC9 mRNA expression and plasma HDAC9 levels. (XLSX)

S1 Fig HRM and sequencing analyses for different genotypes of SNP rs2107595. (A)HRM plots for different genotypes of SNP rs2107595. The normalized melting peaks are given in the left column, and the normalized melting curves are given in the right column. Arrows indicate the genotypes. Heterozygous samples are identified by a change in melting curve shape, and different homozygotes are distinguished by melting temperature (Tm) shifts;(B) Direct sequencing analyses for different genotypes of SNP rs2107595.
(TIFF)

S1 Table Primer details and PCR conditions for HRM, direct sequencing and RT-qPCR analyses in our study. (DOCX)

S2 Table Clinical characteristics of participants in our study. (DOCX)

S3 Table Comparative analyses for clinical and genetic characteristics between the randomly selected subjects and the whole samples. (DOCX)
