# Supplementary material for: HDAC9 Variant Rs2107595 Modifies Susceptibility to Coronary Artery Disease and the Severity of Coronary Atherosclerosis in a Chinese Han Population
Source: PLoS One. 2016 Aug 5;11(8):e0160449. doi: 10.1371/journal.pone.0160449 (PMC4975504; doi:10.1371/journal.pone.0160449)
Supplement: S1 Appendix — (DOCX) [file pone.0160449.s001.docx]

**Supplementary materials and methods**

**Diagnostic criterion of different CAD subtypes**

Stable angina pectoris (SAP) was defined as angiographically confirmed coronary artery disease (CAD), and no change in frequency, duration, or intensity of chest pain in at least 2 months at the time of enrollment. Unstable angina pectoris (UAP) was defined as angina of increasing frequency with less exertion and/or at rest or nocturnal and/or severe or prolonged episodes associated with electrocardiographic changes or evidence of ischemia, with negative changes of cardiac biomarkers (creatine kinase MB (CK-MB) and cardiac troponin I (cTnI)) at the time of enrollment. Non-ST-segment elevation myocardial infarction (NSTEMI) was defined as symptoms with clinical presentation of acute coronary syndrome, with elevated levels of cardiac biomarkers, without ST-segment elevation on electrocardiogram. ST-segment elevation myocardial infarction (STEMI) was defined as symptoms with clinical presentation of acute coronary syndrome, with chest pain lasting ≥ 1 mm in at least 2 contiguous leads on a 12-lead electrocardiogram.

**Definition of clinical characteristics**

Individuals who smoked ≥ 100 cigarettes in their lifetime were defined as “smokers”, which included ever smokers and current smokers. An ever smoker was a person who had quit smoking at least 1 year prior to interview. Subjects with alcohol consumption at least once a week for ≥ 1 year were defined as “alcohol drinkers”. One drink was defined as 375 ml of beer (13.6 g of ethanol), 118 ml of wine (11.7 g of ethanol), or 30 ml of western or Chinese hard liquor (10.9 g of ethanol). Hypertension was defined as ongoing therapy for hypertension, systolic blood pressure (SBP) of ≥ 140mmHg or diastolic blood pressure (DBP) of ≥ 90mmHg. Type 2 diabetes mellitus (T2DM) was defined as ongoing therapy for diabetes or fasting plasma glucose (FPG) levels of ≥ 7.0 mmol/L, or plasma glucose levels of ≥ 11.1 mmol/L, or a 2-h plasma glucose level of ≥ 11.1 mmol/L during an oral glucose tolerance test. Hyperlipidemia was defined as hypercholesterolemia (serum TC > 5.72 mmol/L), high levels of LDL-c (> 3.1 mmol/L), low levels of HDL-c (< 0.9 mmol/L), hypertriglyceridemia (serum TG > 1.70 mmol/L).
