# Supplementary figures and images for: HDAC9 Variant Rs2107595 Modifies Susceptibility to Coronary Artery Disease and the Severity of Coronary Atherosclerosis in a Chinese Han Population
Source: PLoS One. 2016 Aug 5;11(8):e0160449. doi: 10.1371/journal.pone.0160449 (PMC4975504; doi:10.1371/journal.pone.0160449)

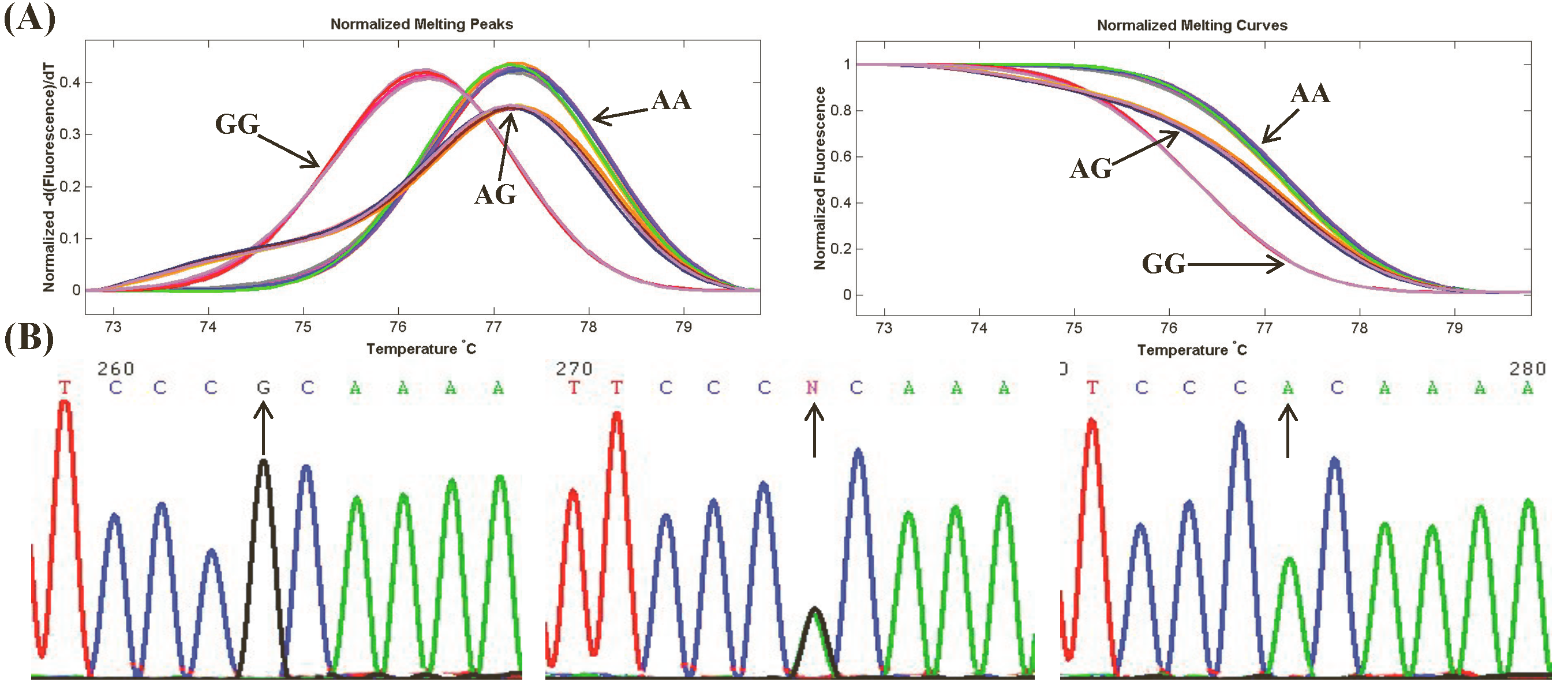

Supplement: S1 Fig — (A)HRM plots for different genotypes of SNP rs2107595. The normalized melting peaks are given in the left column, and the normalized melting curves are given in the right column. Arrows indicate the genotypes. Heterozygous samples are identified by a change in melting curve shape, and different homozygotes are distinguished by melting temperature (Tm) shifts;(B) Direct sequencing analyses for different genotypes of SNP rs2107595. (TIFF) [file pone.0160449.s004.tiff]
