# Supplementary material for: HDAC9 Variant Rs2107595 Modifies Susceptibility to Coronary Artery Disease and the Severity of Coronary Atherosclerosis in a Chinese Han Population
Source: PLoS One. 2016 Aug 5;11(8):e0160449. doi: 10.1371/journal.pone.0160449 (PMC4975504; doi:10.1371/journal.pone.0160449)
Supplement: S1 Table — (DOCX) [file pone.0160449.s005.docx]

| **S1 Table. Primer details and PCR conditions for HRM, direct sequencing and RT-qPCR analyses in our study.** | | | | |
| --- | --- | --- | --- | --- |
| Method | Variables | Primers | Length (bp) | Ann. Temp (°C) |
| HRM * | SNP rs2107595 | Forward: TCTTTTTTGTGTGCTTGTACATTCT | 59 | 57 |
|  |  | Reverse: ATTACTGTGGGACAAAAACATTTTC |  |  |
| Sequencing † | SNP rs2107595 | Forward: ATTGCATCCAGGAACTCA | 477 | 54 |
|  |  | Reverse: TAATGCTTGGCAGGAACA |  |  |
| RT-qPCR ‡ | *TXNIP* | Forward: AGGTGCGGTCCAGGTTAA | 172 | 57 |
|  |  | Reverse: GGCCCATTGTTTGGTGAA |  |  |
|  | *GAPDH* | Forward: GAAGGTGAAGGTCGGAGTC | 226 | 57 |
|  |  | Reverse: GAAGATGGTGATGGGATTTC |  |  |
| * PCR reaction for HRM was performed in a volume of 10 μL containing 25 ng of genomic DNA, 5 pmol of each primer, 2 mmol dNTPs, 2 μL of 10×PCR buffer with 1.5 mmol/L MgCl_2_, 1 unit of Taq polymerase and 1 μL of LC green. The cycling conditions for HRM analyses are 95 °C for 5 min followed by 45 cycles of 20 s at 95 °C, 20 s at 57 °C and 30 s at 72 °C, and final extensions of 30 s at 95 °C and 30 s at 28 °C for heteroduplex formation.  † The cycling conditions for sequencing are 95 °C for 5 min, 35 cycles of 95 °C for 30 s, 30s at 54 °C and 45 s at 72 °C.  ‡ RT-qPCR analysis was performed in a final volume of 20 μL reaction mixture containing 10 μL of 1 X SYBR Green Master mix, 5 pmol of each primer and 50 ng cDNA products. The cycling conditions for RT-qPCR are 95 °C for 5 min and 40 cycles of 95 °C for 15 s and 57 °C elongation for 45s.  HRM, High Resolution Melt; Ann. Temp., annealing temperature; RT-qPCR, reverse-transcription quantitative PCR. | | | | |
