# Supplementary material for: HDAC9 Variant Rs2107595 Modifies Susceptibility to Coronary Artery Disease and the Severity of Coronary Atherosclerosis in a Chinese Han Population
Source: PLoS One. 2016 Aug 5;11(8):e0160449. doi: 10.1371/journal.pone.0160449 (PMC4975504; doi:10.1371/journal.pone.0160449)
Supplement: S2 Table — (DOCX) [file pone.0160449.s006.docx]

| **S2 Table. Clinical characteristics of participants in our study.** | | | | | | | | | | | |
| --- | --- | --- | --- | --- | --- | --- | --- | --- | --- | --- | --- |
| Variables | Discovery set (Study 1) | | |  | Replication set (Study 2) | | |  | Merged set (Study 1 + Study 2) | | |
|  | CAD (N = 1172) | Controls (N = 1086) | P * |  | CAD (N = 1145) | Controls (N = 1318) | P * |  | CAD (N = 2317) | Controls (N = 2404) | P * |
| Age, years | 62.4 ± 9.7 | 62.6 ± 10.5 | 0.674 |  | 63.0 ± 9.6 | 62.6 ± 10.9 | 0.322 |  | 62.7 ± 9.6 | 62.6 ± 10.7 | 0.714 |
| Male, n (%) | 655 (55.9) | 591 (54.4) | 0.484 |  | 623 (54.4) | 732 (55.5) | 0.575 |  | 1278 (55.2) | 1323 (55.0) | 0.932 |
| BMI, kg/m^2^ | 25.2 ± 3.8 | 24.1 ± 2.1 | < 0.001 |  | 25.1 ± 4.0 | 24.1 ± 2.2 | < 0.001 |  | 25.1 ± 3.9 | 24.1 ± 2.1 | < 0.001 |
| Smoking, n (%) | 393 (33.5) | 296 (27.3) | 0.001 |  | 412 (36.0) | 373 (28.3) | < 0.001 |  | 805 (34.7) | 669 (27.8) | < 0.001 |
| Alcohol drinking, n (%) | 368 (31.4) | 257 (23.7) | < 0.001 |  | 389 (34.0) | 328 (24.9) | < 0.001 |  | 757 (32.7) | 585 (24.3) | < 0.001 |
| Hypertension, n (%) | 731 (62.4) | 409 (37.7) | < 0.001 |  | 643 (56.2) | 487 (36.9) | < 0.001 |  | 1374 (59.3) | 896 (37.3) | < 0.001 |
| T2DM, n (%) | 378 (32.3) | 279 (25.7) | 0.001 |  | 372 (32.5) | 335 (25.4) | < 0.001 |  | 750 (32.4) | 614 (25.5) | < 0.001 |
| Hyperlipidemia, n (%) | 347 (29.6) | 242 (22.3) | < 0.001 |  | 334 (29.2) | 307 (23.3) | 0.001 |  | 681 (29.4) | 549 (22.8) | < 0.001 |
| SBP, mmHG | 149.0 ± 34.6 | 131.3 ± 19.1 | < 0.001 |  | 149.2 ± 35.3 | 133.1 ± 39.1 | < 0.001 |  | 149.1 ± 34.9 | 132.3 ± 31.7 | < 0.001 |
| DBP, mmHG | 90.7 ± 17.9 | 82.1 ± 11.8 | < 0.001 |  | 91.1 ± 18.2 | 82.9 ± 12.0 | < 0.001 |  | 90.9 ± 18.1 | 82.5 ± 11.9 | < 0.001 |
| FPG, mmol/L | 5.68 ± 1.68 | 5.38 ± 1.64 | < 0.001 |  | 5.72 ± 1.74 | 5.29 ± 1.64 | < 0.001 |  | 5.70 ± 1.71 | 5.33 ± 1.64 | < 0.001 |
| TC, mmol/L | 5.01 ± 1.01 | 4.79 ± 0.93 | < 0.001 |  | 5.02 ± 1.00 | 4.82 ± 0.93 | < 0.001 |  | 5.02 ± 1.01 | 4.81 ± 0.93 | < 0.001 |
| TG, mmol/L | 1.62 ± 0.90 | 1.39 ± 0.78 | < 0.001 |  | 1.66 ± 0.89 | 1.40 ± 0.78 | < 0.001 |  | 1.64 ± 0.89 | 1.39 ± 0.78 | < 0.001 |
| LDL-c, mmol/L | 3.01 ± 0.93 | 2.76 ± 0.72 | < 0.001 |  | 2.93 ± 0.92 | 2.77 ± 0.77 | < 0.001 |  | 2.97 ± 0.93 | 2.76 ± 0.75 | < 0.001 |
| HDL-c, mmol/L | 1.01 ± 0.16 | 1.26 ± 0.21 | < 0.001 |  | 1.01 ± 0.17 | 1.24 ± 0.21 | < 0.001 |  | 1.01 ± 0.16 | 1.25 ± 0.21 | < 0.001 |
| CAD subtypes, n (%) |  |  |  |  |  |  |  |  |  |  |  |
| SAP | 353 (30.1) |  |  |  | 327 (28.6) |  |  |  | 680 (29.3) |  |  |
| UAP | 400 (34.1) |  |  |  | 376 (32.8) |  |  |  | 776 (33.5) |  |  |
| NSTEMI | 272 (23.2) |  |  |  | 271 (23.7) |  |  |  | 543 (23.4) |  |  |
| STEMI | 147 (12.6) |  |  |  | 171 (14.9) |  |  |  | 318 (13.7) |  |  |
| Modified Gensini score | 30.0 (19.5-67.5) | - |  |  | 31.0 (19.5-78.0) | - |  |  | 30.0 (19.5-72.0) | - |  |
| * For continuous variables, normally distributed data are expressed as mean ± standard deviation (SD), while skewed data are described as median (interquartile range). For categorical, data are expressed as frequency counts.  CAD, coronary artery disease; BMI, body mass index; T2DM, type 2 diabetes mellitus; SBP, systolic blood pressure; DBP, diastolic blood pressure; FPG, fasting plasma glucose; TC, total cholesterol; TG, triglyceride; LDL-c, low-density lipoprotein cholesterol; HDL-c, high-density lipoprotein cholesterol; SAP, stable angina pectoris; UAP, unstable angina pectoris; NSTEMI: non-ST-segment elevation myocardial infarction; STEMI: ST-segment elevation myocardial infarction.. | | | | | | | | | | | |
