# Supplementary material for: HDAC9 Variant Rs2107595 Modifies Susceptibility to Coronary Artery Disease and the Severity of Coronary Atherosclerosis in a Chinese Han Population
Source: PLoS One. 2016 Aug 5;11(8):e0160449. doi: 10.1371/journal.pone.0160449 (PMC4975504; doi:10.1371/journal.pone.0160449)
Supplement: S3 Table — (DOCX) [file pone.0160449.s007.docx]

| **S3 Table. Comparative analyses for clinical and genetic characteristics between the randomly selected subjects and the whole samples.** | | | | | | | |
| --- | --- | --- | --- | --- | --- | --- | --- |
| Variables * | Control | | |  | CAD | | |
|  | Selected subjects (N = 125) | Whole samples (N = 2404) | P † |  | Selected subjects (N = 125) | Whole samples (N = 2317) | P † |
| Age, (year) | 63.1 ± 10.6 | 62.6 ± 10.7 | 0.645 |  | 64.0 ± 10.4 | 62.7 ± 9.6 | 0.142 |
| Male, n (%) | 71 (56.8) | 1323 (55.0) | 0.699 |  | 66 (52.8) | 1278 (55.2) | 0.606 |
| BMI, kg/m^2^ | 24.0 ± 2.2 | 24.1 ± 2.1 | 0.779 |  | 25.7 ± 4.4 | 25.1 ± 3.9 | 0.120 |
| Smoking, n (%) | 33 (26.4) | 669 (27.8) | 0.728 |  | 51 (40.8) | 805 (34.7) | 0.167 |
| Drinking, n (%) | 31 (24.8) | 585 (24.3) | 0.906 |  | 42 (33.6) | 757 (32.7) | 0.829 |
| Hypertension, n (%) | 48 (38.4) | 896 (37.3) | 0.799 |  | 78 (62.4) | 1374 (59.3) | 0.492 |
| T2DM, n (%) | 33 (26.4) | 614 (25.5) | 0.830 |  | 42 (33.6) | 750 (32.4) | 0.775 |
| Hyperlipidemia, n (%) | 31 (24.8) | 549 (22.8) | 0.611 |  | 37 (29.6) | 681 (29.4) | 0.960 |
| Rs2107595, n (%) |  |  | 0.415 |  |  |  | 0.131 |
| GG | 65 (52.0) | 1158 (48.2) |  |  | 50 (40.0) | 976 (42.1) |  |
| AG | 46 (36.8) | 1023 (42.6) |  |  | 54 (43.2) | 1087 (46.9) |  |
| AA | 14 (11.2) | 223 (9.3) |  |  | 21 (16.6) | 254 (11.0) |  |
| CAD subtypes, n (%) |  |  |  |  |  |  | 0.204 |
| SAP |  |  |  |  | 45 (36.0) | 680 (29.3) |  |
| UAP |  |  |  |  | 34 (27.2) | 776 (33.5) |  |
| NSTEMI |  |  |  |  | 25 (20.0) | 543 (23.4) |  |
| STEMI |  |  |  |  | 21 (16.8) | 318 (13.7) |  |
| Modified Gensini score |  |  |  |  | 30.0 (18.0-78.5) | 30.0 (19.5-72.0) | 0.394 |
| * For continuous variables, normally distributed data are expressed as mean ± SD, while skewed data are described as median (interquartile range). For categorical, data are expressed as frequency counts.  † The Pearson χ^2^ test and the student t-test (or the Mann-Whitney U test) were used to test for categorical variables and continuous variables, respectively.  N, number; CAD, coronary artery disease; BMI, body mass index; T2DM, type 2 diabetes mellitus; SAP, stable angina pectoris; UAP, unstable angina pectoris; NSTEMI: non-ST-segment elevation myocardial infarction; STEMI: ST-segment elevation myocardial infarction.. | | | | | | | |
